# The Microbiome and Sustainable Healthcare

**DOI:** 10.3390/healthcare3010100

**Published:** 2015-03-03

**Authors:** Rodney R. Dietert, Janice M. Dietert

**Affiliations:** 1 Department of Microbiology and Immunology, College of Veterinary Medicine, Cornell University, Ithaca, NY 14853, USA; 2 Performance Plus Consulting, Lansing, NY 14882, USA; E-Mail: SonofStar@aol.com

**Keywords:** non-communicable diseases, economic burden, gut microbiota, personalized medicine, antibiotic resistance, microbiome reconstitution, pregnancy, archaea, bacteria, immune maturation

## Abstract

Increasing prevalences, morbidity, premature mortality and medical needs associated with non-communicable diseases and conditions (NCDs) have reached epidemic proportions and placed a major drain on healthcare systems and global economies. Added to this are the challenges presented by overuse of antibiotics and increased antibiotic resistance. Solutions are needed that can address the challenges of NCDs and increasing antibiotic resistance, maximize preventative measures, and balance healthcare needs with available services and economic realities. Microbiome management including microbiota seeding, feeding, and rebiosis appears likely to be a core component of a path toward sustainable healthcare. Recent findings indicate that: (1) humans are mostly microbial (in terms of numbers of cells and genes); (2) immune dysfunction and misregulated inflammation are pivotal in the majority of NCDs; (3) microbiome status affects early immune education and risk of NCDs, and (4) microbiome status affects the risk of certain infections. Management of the microbiome to reduce later-life health risk and/or to treat emerging NCDs, to spare antibiotic use and to reduce the risk of recurrent infections may provide a more effective healthcare strategy across the life course particularly when a personalized medicine approach is considered. This review will examine the potential for microbiome management to contribute to sustainable healthcare.

## 1. Introduction

Healthcare is facing two serious challenges that appear to be inter-related. The first is the significant rise in prevalence of multiple non-communicable diseases and conditions (NCDs) (e.g., asthma, food allergies, obesity, celiac disease, type 1 diabetes, type 2 diabetes, inflammatory bowel disease, autism, Alzheimer’s disease, Parkinson’s disease, heart disease and cancer) not just in developed countries but globally. These diseases already account for a majority of deaths worldwide and are expected to continue to increase in impact in the coming decades [1]. To date, most NCDs are not cured but rather are medically managed across a lifetime often at considerable cost and with reduced quality of life.

This is paired with a second challenge: the emergence of multi-drug resistant bacterial pathogens that are outpacing the discovery and production of new antibiotics [2]. Multi-drug resistant bacteria are thought to have arisen in part due to the overuse of antibiotics [3]. These two challenges are pressuring healthcare in terms of: (1) the effectiveness of treatments, (2) the level of care required and (3) the global economic resources required to treat these diseases. However, a solution to these challenges may reside within the patients themselves, specifically, within their microbiomes.

Over the past decade, multi-disciplinary research on the microbiome has brought forward a completely new view of what it means to be human, and this view is beginning to affect the direction of healthcare [4,5]. Research findings indicate that humans have approximately 10-times the number of microbial cells as mammalian cells and that our majority microbial genes drive fundamental human biological processes affecting virtually every organ and physiological system [6,7,8,9,10,11]. In fact, the microbial genetic component of humans is sufficiently significant that it has been referred to as our “second genome” [12]. This is forcing aside what had been a purely mammalian-centric view of the human patient and replacing it with something quite different: humans as holobionts [13].

**Figure 1:**
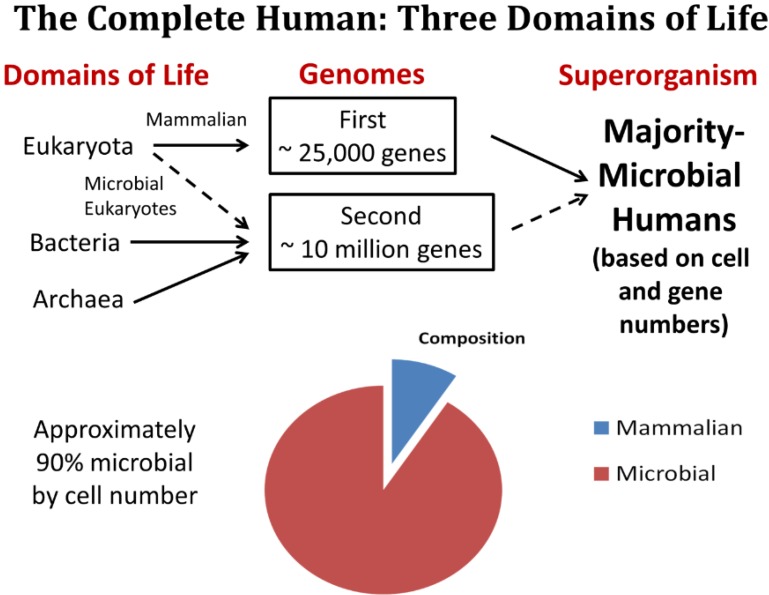
The Three-Domain Model of humans is depicted. It is estimated that we are approximately 90% microbial in composition (by number of cells) in our healthiest state with approximately 25,000 gene sequenced from the human genome (the first genome) and almost 10 million genes sequenced from among our microbiota (the second genome).

As recently envisioned, humans in their healthiest state are a complex, mutualistic ecosystem comprised of all three domains of life (Bacteria, Archaea and Eukaryotes) with the majority of cells and genes being microbial (Figure 1). Even within the domain of the Eukaryotes, there is representation in the human microbiome by non-mammalian organisms such as unicellular fungi and others (microbial eukaryotes) [14].

This is all the more remarkable when it is considered that one of our mutualistic partners, the Archaea, have been termed extremophiles in that they can survive and often thrive in earth’s most extreme environments [15,16]. The human immune and other systems need to interact with this diversity of non-mammalian mutualistic organisms for the human to be considered biologically and functionally complete [17,18,19]. If much of medical practice and health management was previously focused on the 10% mammalian portion of the patient to the exclusion of all else, future healthcare is likely to place increasing attention on managing the 90% that is non-mammalian. Integrated healthcare of the human-microbial superorganism is actually a form of ecological management where risk-benefit decisions will include population ecology issues encompassing all three kingdoms of life within the patient [20,21]. A three-domain approach to human health seems likely to affect everything from medical procedures, birth processes, pregnancy and dietary management, applications of personalized medicine, drug design and therapies, end-of-life care, and safety evaluation.

Redefinition of the human biological constitution and associated redirection of healthcare can be viewed as positive factors in the struggle to combat the global epidemic of multiple NCDs and the increasing threat of antibiotic resistant bacterial pathogens. In fact, in this mini-review we argue that: (1) recent microbiome information is spawning a useful sea change in medical approaches, and that (2) the rapidly-shifting approach to human health management directed at the human holobiont has the potential not only to blunt ongoing epidemics and pandemics but also to bring personalized medicine to its full potential and set us on a path toward sustainable healthcare.

## 2. The Epidemic of Non-Communicable Diseases

One of the significant threats to healthcare systems, if not the economic stability of entire countries, appears to be the ongoing epidemic of NCDs [1,22]. NCDs can arise in different tissues or organs, or alternatively, become systemic diseases. They fall into various categories often designated as allergic, autoimmune, inflammatory, metabolic, neurobehavioral or neurodegenerative. These include: allergic rhinitis, Alzheimer’s disease, asthma, atopic dermatitis, autism, autoimmune thyroiditis, cancer (numerous manifestations), cardiovascular disease (several manifestations), celiac disease, chronic kidney disease, diabetes (both type 1 and type 2), dementia, depression, food allergies, inflammatory bowel disease, multiple sclerosis, obesity, Parkinson’s disease, psoriasis, schizophrenia, systemic lupus erythematosus, systemic sclerosis, rheumatoid arthritis, sensory loss (various), and sleep disorders (several). Unlike infectious diseases that may be either transient, latent, or result in rapid death, NCDs are often maintained over a longer duration of life.

Living with an NCD impacts quality of life, increases healthcare requirements, and often results in premature death. In fact measures such as disability adjusted life years (years living with a disease-associated disability) appear to be one of the most significant metrics impacted by the epidemic of NCDs [23]. Disease management often involving intensive medical care, drug requirements and potential caregiver help has been the standard approach, to date, for most NCDs. In the past NCDs were generally thought to be the purview of Westernized more developed countries, but they are now a global problem stressing healthcare systems [24,25].

The World Health Organization Global Monitoring Framework has established a list of NCDs for reduced prevalence by 2025 [26]. The goal is stated under the phrase 25 by 25 meaning that prevalence of premature mortality due to NCDs should be reduced by 25% (compared against 2010 levels) by the year 2025 [27]. This focus of the framework is on the probability of dying between the ages of 30–70 from cardiovascular disease, cancer, diabetes or chronic respiratory diseases. Four behavioral risk factors were identified: harmful use of alcohol, physical inactivity, salt/sodium intake, and tobacco use [27].

However, the focus on the four lifestyle factors fails to incorporate the findings of two recent, emerging fields of study: (1) microbiome studies in which humans are recognized and treated as if they are majority microbial rather than solely mammalian [28], and (2) studies emphasizing the developmental origins of adult health and disease (DOHaD) and their impact on lifetime health [29,30]. The intersection of these two areas of study is beginning to set a template for future healthcare strategies [18,31]. Recently, Collado *et al*. [32] suggested that a focus on the microbiota particularly during early life might be an effective disease prevention strategy. This has been facilitated by a recent intensive research effort on the microbiome including the recent multi-country cataloging of nearly 10 million intestinal microbial reference genes in humans [33].

**Figure 2:**
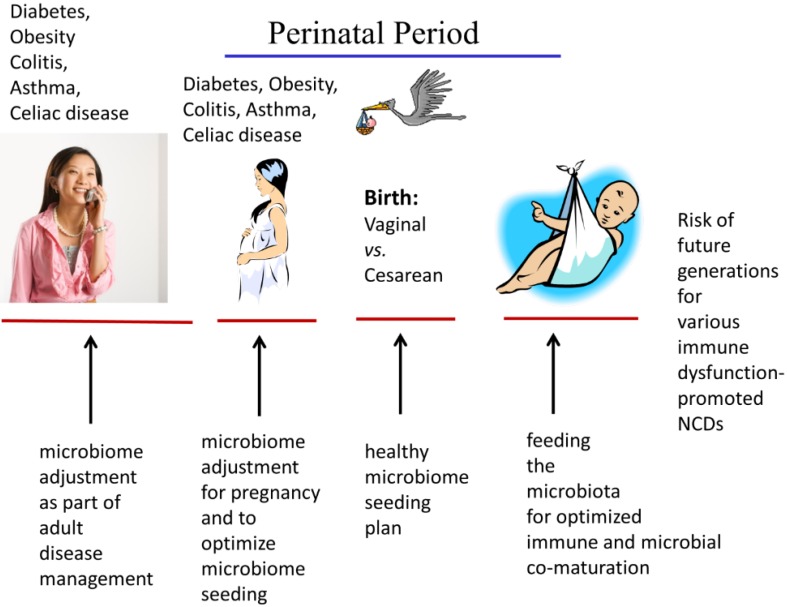
Life stage-based approaches to microbiome management are depicted beginning with the young adult and following through pregnancy and birth + nurturing of the next generation. Considerations for each life stage are illustrated such as the preparation, seeding and feeding of the infant microbome. The perinatal period of development is a particularly important window for potential modifications that could positively impact immune maturation and the risk of immune-related and inflammatory-driven disorders.

While there are opportunities for microbiome management in patients of any age, certain key life stages warrant special attention. Figure 2 illustrates the potential opportunities for microbiome management at different life stages. It particular, the life stage windows of pregnancy, birth, and early childhood provide intervention points where alterations of the microbiome are likely to have lifelong consequences and to fully impact the maturation and developmental programming of the child’s physiological systems (e.g., the immune system, brain).

Table 1 illustrates some of the reported microbial dysbiosis associated with NCDs. Causality remains to be determined for many of these associations, however, in many cases the microbial dysbiosis and associated metabolic changes precede other biomarkers of the NCD. Additionally, immune dysfunction associated with the microbial dysbiosis appears to be directly linked with the improper tissue responses leading to many of these NCDs. As will be discussed in later sections, treatments aimed at correcting gut microbial dysbiosis (termed rebiosis) have shown promise in correcting some of the NCDs [34].

**Table 1:** Microbial dysbiosis and non-communicable diseases and conditions (NCDs).

Non-communicable Diseases and Conditions	Sample/Location	Reference(s)
Asthma	Bronchial and gastrointestinal	[35]
Atopic dermatitis	Skin	[36]
Autism spectrum disorder	Gastrointestinal	[37]
Behcet’s syndrome	Gastrointestinal	[38]
Breast cancer	Breast tissue	[39]
Cardiovascular disease (e.g., atherosclerosis)	Gastrointestinal	[40,41]
Celiac disease	Gastrointestinal	[42]
Chronic kidney disease	Gastrointestinal	[43]
Chronic obstructive pulmonary disease (COPD)	Respiratory	[44]
Chronic periodontitis	Subgingival	[45]
Colorectal cancer	Gastrointestinal	[46,47]
Crohn’s disease	Gastrointestinal	[48,49]
Esophageal squamous cell carcinoma	Upper digestive tract	[50]
Food allergy	Gastrointestinal	[51]
Gastric cancer	Gastric	[52]
Hypertension	Gastrointestinal	[53]
Laryngeal squamous cell carcinoma	Larnyx and Throat	[54]
Liver cirrhosis	Gastrointestinal	[55]
Lung cancer (non-smokers)	Lung	[56]
Multiple sclerosis	Gastrointestinal	[57]
Non-alcoholic fatty liver disease	Gastrointestinal	[58]
Obesity	Gastrointestinal	[59]
Pancreatic cancer	Salivary	[60]
Parkinson’s disease	Gastrointestinal	[61]
Prostate cancer	Gastrointestinal	[62]
Psoriasis	Skin	[63]
Rheumatoid arthritis	Gastrointestinal	[64]
Systemic lupus erythematosus	Gastrointestinal	[65]
Type 1 diabetes	Gastrointestinal	[66]
Type 2 diabetes	Gastrointestinal	[67]
Ulcerative colitis	Gastrointestinal	[68]

## 3. Antibiotics, Microbial Dysbiosis, and Microbiome Insufficiency

The discovery and mass production of antibiotics are among the greatest medical discoveries and breakthroughs of the 20th century [69]. These breakthroughs have saved countless lives [70]. Yet, more of a good thing is not always biologically advantageous, and recent findings provide a more useful view of the risk-benefits of current antibiotic regimes, particularly during early life. Two issues with antibiotic treatments are challenging healthcare. First, overuse has led to an increase in antibiotic-resistant strains of bacteria that limit treatment options, and have, in some cases, created potential hazards [71]. This has led to calls for system-wide public health action [72,73] and resulted in stewardship programs [74] and inventory strategies aimed at targeting the use of antibiotics [75], reducing the pressure on selection of antibiotic resistance, and eventually improving the hospital environment in terms of risk-benefit [76].

There has been a call for multifaceted therapeutic interventions to reduce the overuse of antibiotics particularly when treating respiratory symptoms (the majority of prescriptions by general practitioners) [77]. Acute otitis media (AOM) is one of the conditions where antibiotic administration has been routine. However, the risk-benefits of this approach has been called into question when considered in light of the risk of adverse outcomes following antibiotic administration, spontaneous remission of AOM without complications, and the need to preserve the effectiveness of antibiotics for more serious conditions [78,79]. To date, in hospitals where some combination therapies have been given a higher priority, the results are mixed [2]. Recent recommendations have encouraged a more restricted use of antibiotics, at least among developed countries. These include a policy of prohibiting over-the counter sales of antibiotics, broader implementation of rapid point-of-care tests, promotion of strategies that encompass delayed antibiotic prescription, more active participation of clinicians in audits of antibiotic prescriptions, and better information for clinicians on complications and longer-term adverse outcomes of antibiotic use [77].

The impact of antibiotics on microbial diversity and selection of drug-resistant pathogens is not restricted only to use in human medicine. Similar restricted-use measures have been advocated for animal agriculture where prophylactic use of antibiotics in animal feed can drive drug-resistance among zoonotic strains of pathogens [80] as well as in veterinary medicine [81].

Second, even appropriate use of antibiotics can have previously unknown, long-term consequences unless complementary measures addressing the integrity of the microbiome are pursued. Administration of antibiotics affects not only bacterial pathogens but also needed microbiota comprising the microbiome. Given that our mutualistic microbiota have been regarded biologically as a hidden and sometimes missing organ [82,83], an inherent side effect of antibiotic administration is reduced presence and decreased function of our microbiome “organ” and a cascade of altered functions in other organs [84].

Antibiotic-induced microbial dysbiosis or microbiome insufficiency disrupts host physiology and defenses via changes in both metabolism [85] and immune function [86]. This occurs when antibiotic treatment eliminates commensal bacteria that were producing needed metabolic and physiological signals for host systems such as the brain, immune, and digestive systems. Antibiotic-induced elimination of useful microbiota also increases the risk for recurrent infections as a longer term adverse consequence [87,88]. Pregnancy and the neonatal period of life are particularly critical relative to the adverse, long term implications of antibiotic administration [89].

Microbiome reconstitution/rebiosis, whether through targeted probiotic-live culture administration [84] or more comprehensive fecal microbiota transplantation, is a new area that shows promise in alleviating the adverse impact to the microbiome of antibiotic administration [90,91]. This is discussed in more detail in Section 7 on Microbiome Management. While challenges exist, complementary therapies designed to reseed and/or restore the patient’s microbiome appear likely to reduce the long-term risks associated with antibiotic therapies [92].

## 4. The Microbiome as a New Focus of Healthcare

The microbiome is largely situated at the portals of entry, which are directly exposed to our external environment (e.g., skin, gastrointestinal tract, airways, and urogenital tract). As a result, our microbiota are the first exposed and the first responders to food, air, water, and drugs [19,93]. Individual factors are handled differently by different microbial species affecting the overall metabolism and sensitivity to different external factors. When this is combined with the recognition that the microbiome can regulate the maturation and function of numerous physiological systems, effective management of the microbiome takes a significantly higher priority.

Metabolism via the microbiome is a critical factor in the development and/or maintenance of certain disease states. For example metagenomic analysis of the gut microbiome has revealed important differences among obese *vs.* non obese individuals. Greenblum *et al*. [94] reported that both obesity and inflammatory bowel disease are associated with important differences in the way the disease-associated gut microbiome interacts with host metabolism. Additionally, there is some evidence that disease profiles can be transferred with transplantation of skewed, disease-supporting microbiota. Ridaura *et al*. [95] found that when the microbiota from obese *vs.* lean human twins were transplanted into adult male C57/BL6/J6 germ-free mice, the mice acquired the same functional metabolic profile as their human donor. For example, mice receiving a lean *vs.* obese microbiome transplantation had a higher level of gene expression for genes involved in digestion of plant-derived polysaccharides. Co-housing of the recipient mice could phenotypically rescue the ones given the obese-associated microbiome.

Microbiome management encompasses all aspects of seeding, feeding, protection, and useful manipulation of the gut, oral, respiratory, dermal, and urogenital microbiota. Both microbial fingerprinting and metabolonomic analyses are likely to play an increasing role in medical management of the microbiome. An important contribution has been the effort of Eggesbø *et al*. [96] to map the transitions among gut microbiota that occur during normal human development in the absence of medical interventions. This provides a needed standard against which age-matched, disease-associated profiles may be compared.

A wide variety of factors has been reported to affect the microbiome. These include lifestyle choices, specific medical procedures (e.g., Cesareran delivery (CD)), diet, infections, environmental chemicals (e.g., heavy metals and endocrine disruptors), drugs, and stress. Examples of these reported associations are shown in Table 2 including the alterations to the microbiome and the reported health effects. Among the studies in Table 2 are reports associating Cesarean delivery with both skewed gut microbiota and elevated risks of a variety of chronic immune disorders. Similarly, early antibiotic use has been associated with an elevated risk of childhood asthma. In a longitudinal birth cohort study, Hoskin-Parr *et al*. [97] found that antibiotic use during the first two years of life was associated with an elevated risk of asthma by 7.5 years of age (OR 1.75, 95% CI 1.40–2.17). A dose dependent effect was also noted associated with the number of rounds of antibiotics administered during infancy. Environmental and dietary factors are also important. Using a mouse model, Ooi *et al*. [98] showed that vitamin D levels affected both the distribution of gut bacteria as well as the numbers of tolerogenic dendritic cells in the gut. The reduced numbers of tolerogenic dendrictic cells predisposed the mice to inflammatory injury and colitis.

Additionally, using a mouse model for autism spectrum disorders (ASD) (Table 2), de Theije *et al*. [99] showed that in utero exposure to valproate altered neonatal gut microbiota, the production of short chain fatty acids (SCFAs), and the development of autism-like behavior. This is significant for humans since fecal metabolite distinctions have been reported among autistic children [100] and recent evidence suggests that early developmental levels of proprionic acid (produced by enteric bacteria) affect neurodevelopmental behavior [101]. Additional biomarkers related to the elevated proprionic acid production model were studied in ASD children. Associated markers in a subset of these children included altered profiles of long chain acetyl carnitines as well as both altered glutathione metabolism and mitochondrial dysfunction [102]. This is useful in providing a potential mechanistic linkage between microbial dysbiosis and ASD. Nankova *et al*. [103] reported that one of the mechanisms through which the enteric bacteria-produced SCFAs appear to affect neurdevelopment and potential risk of ASD is via epigenetic modulation of cell function. The levels of proprionic acid and butyric acid can affect neurotransmitter-related gene expression as well as oxidative stress, inflammation, lipid metabolism and mitochondrial function. [103].

Finally, also in mice (Table 2), An *et al*. [104] showed that *Bacteroides fragilis* in particular needs to be present in the newborn gut to produce sphingolipids that inhibit a burst of invariant natural killer T cells (iNKT). In the absence of these bacteria and the key metabolites, the mice are programmed for gut inflammation and the development of colitis. This study supports the importance of the perinatal developmental window for holobiont self-completion.

**Table 2:** Medical and various environmental factors reported to affect the microbiome.

Category	Factor	Evaluation System	Reported Effect on Microbiota	Reported Health Effect	Ref(s)
Medical	Cesarean delivery (CD)	Human	Reduced microbiota diversity and numbers in the gut	Increased risk of both type 1 diabetes and asthma after CD and celiac disease after elective CD	[105,106,107,108,109]
Medical	Fecal microbiota transplants	Human	Increased microbial diversity with increased proportion of *Lachnospiraceae* to *Enterobacteriaceae*; Increase in butyrate-producing bacteria	Protective against recurrent *Clostridium difficile* infections	[110,111,112]
Medical	Infant antibiotic use	Human	Reduced diversity among bifidobacteria	Increased risk of elevated childhood body mass index (boys); celiac disease, and asthma related to number of antibiotic courses	[97,113,114,115]
Medical	*Bacteroides fragilis* sphingoplipid administration	Mouse	Broader diversity of bacterial metabolites	Reduced numbers of invariant natural killer T cells (iNKT) ; reduced risk of induced autoimmune colitis	[104]
Medical	Tigecycline	Mouse	Antibiotic used to treat *Clostridium difficile* infection; reduced diversity of the microbiota; decreasing *Bacteroidetes*	Treatment increased future susceptibility to *Clostridium difficile* infection	[116]
Medical	Valproate	Mouse	Altered neonatal gut microbiota and butyrate production levels	Elevated risk of autism spectrum disorders	[99]
Dietary	Acidification of liquids in the neonate	Mouse/non-obese diabetic (NOD)	Lowered gut pH; altered gut microbiota species	Reduced risk of diabetes	[117]
Dietary	Aspertame	Rat (Sprague-Dawley males)	Altered gut microbiota with increased production of proprionic acid	Elevated glucose levels and impaired insulin-stimulated glucose disposal (a test for insulin tolerance capacity)	[118]
Dietary	L-Carnitine	Human	Metabolism to trimethylamine by special oxygenases of human microbiota	Some studies report promotion of cardiovascular disease	[41,119]
Dietary	Low dietary fiber content	Mouse	Altered gut microbiota distribution with lower levels of short chain fatty acids produced	Increased allergic airway inflammation	[120]
Dietary and Environmental	Reduced Vitamin D	Mouse C57BL/6 and Human	Increased gut *Helicobacteraceae* family member numbers with increased inflammation	Increased risk of colitis	[98,121]
Environmental	Arsenic exposure	Mouse/C57/BL6	Gut microbiota affects arsenic metabolism which, in turn, alters the abundance and composition of the microbiota	Elevated risk of cardiovascular disease	[122,123,124]
Environmental	Cadmium exposure	Mouse and Human	Reduced abundance of gut microbiota with bacteroides and lactobaccili	Renal dysfunction and increased risk of osteoporosis in humans	[125,126,127,128]
Environmental	Chlorpyriphos exposure	Rat and Human simulation	Altered ratios of microbiota	Increased risk of depression	[129,130]
Environmental	Lead exposure	Mouse/Human	Lower genus diversity of microbes in the gut	Elevated risk of metabolic syndrome, cardiovascular disease, and cognitive impairment	[125,131,132,133]
Environmental	Particulate Matter (PM_10_)	Mouse Wild-type 129/SvEv	Ingestion alters gut microbiota and induces oxidative proinflammation response	Increased risk of cardiovascular disease and asthma	[134,135,136]
Environmental	Polychlorinated biphenyls	Mouse C57BL/6 and Human	Reduced gut microbiota abundance and decreased Proteobacteria	Elevated risk of vaccine failure and allergic sensitization	[137,138,139]
Psychosocial	Stress	Human	Reduced numbers of Lactobacilli with increased gram-negative pathogens	Elevated risk of intestinal disorders including loss of barrier function	[140]
Psychosocial	Stress	Mouse CD-1 males (some effects are strain specific)	Reduced abundance of Bacteroides with increased abundance of Clostridia	Elevated production of innate immune cell pro-inflammatory mediators	[141,142]

## 5. The Microbiome Facilitates a Systems Biology Approach to Healthcare

Recently, researchers and clinicians have called for a systems biology-type of approach to managing NCDs [143]. A systems approach has the advantages of avoiding the pattern in which clinicians are forever pursuing an ever increasing array of symptoms and co-morbid chronic conditions often without a larger context for addressing and/or correcting the root dysbioses that drive NCDs. Aw and Fukuda [144] argue that by integrating metagenomic and metabolomic information across the microbial and mammalian components of the human superorganism, one can better design new therapeutics and integrated treatment strategies. A similar conclusion was reached by Dinan *et al*. [145] who indicated it is time for the microbial genome to be given full consideration when considering the overall genomics linked with schizophrenia. Mao and Frank [146] suggest that by incorporating the microbiome into a systems biology-type of approach at the individual patient level, it is easier to extend effective healthcare and, in particular, useful preventative strategies to the population level.

An important consideration of this approach is the focus on the first 1000 days of life (conception to infancy) as a period where epigenetic programming can exert a tremendous influence over the lifecourse. This has led researchers to call for better integration of human gut microbiota information with an epigenetic platform [147].

## 6. The Microbiome in NCD Co-Morbidities and Increased Disease Vulnerabilities

Microbiome fingerprinting has several advantages in health risk reduction. It can indicate when environmental conditions have resulted in microbial dysbiosis that may need medical attention. But, additionally, it provides a possible therapeutic target to interrupt what is a known progression of highly predictable, immune dysfunction-driven, co-morbid NCDs [148,149]. As shown in Table 1 and Table 2, dysbiosis of the microbiota, at a minimum, helps to maintain the NCD disease state including the misregulation of inflammation in affected tissues. In some cases, it may be causative. Additional evidence suggests that the altered metabolism and physiology of microbial dysbiosis creates additional vulnerabilities that, if unchecked, result in elevated health risks. Several environmental and lifestyle factors affect gut microbiota creating a microbiome fingerprint associated with NCDs such as obesity. Once in place, the altered microbiome can also affect how the individual will interact with the environment (e.g., diet, environmental toxicants) and this can create additional health risks. For example, if exposure to heavy metals including arsenic can alter the microbiome in a manner that supports obesity, the resulting microbiome and disease condition can make the individual more susceptible to subsequent exposure to air pollutants and arsenic via altered chemical handling and metabolism (see also [150]). As a result, microbiome therapy/rebiosis can have a dual benefit in that it may not only interrupt the cycle of co-morbid NCDs but also has the potential to reduce the vulnerability of the host to potentially problematic environmental exposures.

There is evidence of a close relationship between gut microbiota distribution, microbial metabolism and status of immune dysfunction-based disease. Tjellström *et al*. [151] used a fermentation index of short chain fatty acids (SCFAs) to determine gut inflammatory status (the amount of acetic acid minus propionic acid and n-butyric acid, together divided by the total amount of SCFAs) as a means to evaluate the gut microbe status of children with celiac disease who had been on a gluten free diet for more than a year. They found that children maintained on a gluten-free diet for more than year had a normalized gut microbe distribution and SCFA metabolism in contrast with those who had consumed a gluten-free diet for less than a year.

## 7. Microbiome Management: Opportunities and Present Limitations

In this mini-review, it is not our intention to comprehensively review the entire microbiome literature but rather to introduce the range of considerations that go into microbiome management within the healthcare setting. A variety of strategies can be used for rebiosis using what is, in effect, a microbial ecology approach. But most are variants on a theme of seeding with specific microbiota, feeding in support of those microbiota, and analyzing microbial population changes and/or clinical outcomes.

### 7.1 General Considerations

The opportunity to alter 90% of humans that is the microbiome as an integrated health strategy represents one powerful, new tool that can improve healthcare efficiency and spare public health resources. Already there are encouraging signs in treating specific NCDs. As recently reviewed by Scott *et al*. [152], several different approaches can be used (e.g., prebiotics, probiotics, fecal microbiota transplantation) that differ in the degree of microbiome alteration and impact. Obieglo *et al*. [153] recently reviewed the use of microbe-based therapies for treatment of allergic airway disease and microbial reconstitution efforts directed toward the gastrointestinal tract have shown promise in treating ulcerative colitis [154], insulin resistance, and type 2 diabetes [155]. One of the strategies in combating obesity/type 2 diabetes is to elevate the representation of butyrate- and other specific SCFA-producing bacteria, which appear to exert useful anti-inflammatory and immunometabolic effects on these NCDs [156,157]. Likewise, MacFabe and collegues [37,103,158] reported results suggesting that gut microbe production of SCFAs may be important in certain neurodevelopmental conditions such as autism spectrum disorders.

There are several facets to management of the microbiome but, ultimately, they fall into three basic categories (discussed below): (1) determination of the most desirable microbiome to support an individual’s health (including the diversity of, amount of and metabolic activity of microbes), (2) analysis of the microbiota colonizing specific areas of interest in an individual (e.g., skin, oral cavity, respiratory tract, gastrointestinal tract, urogenital tract), and (3) modification of the patient’s microbiome to achive effective balance and improved health (also termed rebiosis).

For category 1 (identifying the ideal microbiome), the microbiomes of healthy people can serve as a prototype, but the specific age, sex, and genetic/epigenetic background of the individual patient may also play a role in the choices that are made. To date, most prototype microbiome data have come from comparisons among healthy groups *vs.* specific disease cohorts. Both sequencing [159] and functional metabolism (e.g., carbon sources) [160] approaches have been used. Recently, clear distinctions among patterns of microbial dysbiois have been noted [161]. As databases increase with the inclusion of large scale, multi-country studies, the goal of defining prototype microbiomes should become easier to achieve. Already, general clustering of microbes among healthy *vs.* specific disease cohorts has led to virtual fingerprints that are useful in predicting and/or identifying healthy *vs.* disease-associated patterns. Shifts between these patterns also appear to be promising as one measure of treatment efficacy. However, one present limitation is that general clustering of microbes by health status may not have the level of detail that is most useful in formulating the most effective therapeutics. An effort is underway to increase the state of the art for *in vitro* culture of human gut microbial communities so that this information can better guide microbiome-based medical practice [162].

For category 2 (microbiome analysis), the sampling and type of organismal, molecular, and/or metabolic analysis is important in identifying potential dysbiosis associated with an existing condition or as an early biomarker of specific health risks. Importantly, a fundamental starting point is the recognition that the microbiome is a pivotal, front-line factor in any biomarker-driven environmental health assessment [163]. Because many of the microbiota are not easily cultured from fecal, *etc*. samples, culture-independent high throughput analysis is proving to be a useful approach. These can include a variety of omics-based comparisons including metagenomic sequencing, metatranscriptomics, and metabolomics [164,165]. Most of these are combined with bioinformatic computational tools [166].

For category 3 (microbiome modification/rebiosis), modifications can be made at any time during the life course and may include introduction of specific prebiotics and probiotics or alternatively, fecal transplantion. However, changes during the perinatal period (Figure 2) are particularly promising in that they are more likely to impact infant immune maturation. As described by Gilbert [13], preparation of the infant microbiome begins in earnest during pregnancy when the maternal microbial organisms appear to participate in creating the pregnancy environment itself (e.g., pregnancy related body changes affected by microbial metabolism) as well as in perinatal transfer of microbiota as part of the birth narrative. Modifications during the perinatal window are particularly important for effective immune maturation and reduced risk of immune-related disorders. The mother’s health status can affect the microbiota that will be used to seed the infant. Gosalbes *et al*. [167] reported that mothers with atopic eczema had a specific, less diverse meconium microbiota profile. A similar finding was reported by Hu *et al*. [168] who found that the mother’s diabetes status affected the profile of the meconium microbiota. Further discussion of modification/rebiosis will be considered for prebiotics and probiotics as well as for fecal tranplantion.

### 7.2 Examples of the Use of Prebiotics, Probiotics, and Synbiotics

Prebiotic, probiotic and synbiotic administration can be useful to selectively modify the gut microbiome [169]. Prebiotics are food ingredients that support beneficial bacteria. They are nondigestable in that they are not broken down in the stomach or absorbed in the gastrointestinal tract but, instead, are fermented by gut microbiota and stimulate growth among one or more selective microbial species (e.g., *Bifidobacteria* bacteria) [170]. Examples of prebiotics include: pectin-derived oligosaccharides from agricultureal by-products, fructo-oligosaccarides (e.g., inulin-like prebiotics), galacto-oligosaccharides (e.g., raffinose) and mannan-oligosaccharides (usually derived from the cell wall of yeast). Probiotics are live bacteria given individually or in combination that are administered to seed the gut. Synbiotics are combined pre- and probiotics designed to both seed and feed newly-administered beneficial gut microbes [169]. As more information has emerged regarding prebiotic and specific probiotic interactions, there has been a trend toward greater use of the synbiotic treatment approach.

Adminstration of prebiotics can be beneficial across a broad spectrum of ages although their effectiveness against many infectious diseases and NCDs has yet to be examined in depth. A systematic review of five randomized trials by Lohner *et al*. [171] concluded that administration of prebiotics to healthy infants reduced the incidence of acute infections from 0 to 24 months (rate ratio 0.68; 95% confidence interval 0.61–0.77). Francino [172] reviewed examples in which administration of pre-and probiotics during late pregnancy in families with a history of allergy reduced the risk of atopic disease in infants. At the other end of the age spectrum, prebiotics have been useful in enhancing influenza vaccine reponses in the elderly [173]. Additionally, some prebiotics may be useful as complementary therapy for patients receiving antibiotics [174]. Prebiotics are also important in promoting anti-inflammatory activity as part of the management of NCDs [175]. In a recent meta-analysis involving thirteen trials of obesity-related comorbidities, Beserra *et al*. [176] concluded that administration of prebiotics and synbiotics has a significant positive effect on the levels of total plasma cholesterol, triglycerides, and lipid parameters (*i.e.,* LDL and HDL). Mirmiran *et al*. [177], recently reviewed the utility of functional foods for the management of type 2 diabetes and concluded that prebiotic-based dietary management of patients is an important piece of any comprehensive dietary approach to disease management.

Probiotic administration has been examined for both disease prevention and improved management. As an example of its use in disease prevention, Deshpande *et al*. [178] reported in a meta-analysis that probiotic administration to preterm neonates significantly reduced dealth and disease from necrotizing enterocolitis. Using pyrosequencing techniques, Zhang *et al*. [179] reported that a 28 day feeding regimen of a particular *Lactobacillus* probiotic resulted in major shifts in both microbiota diversity and representation. Seeding of the new bacterium was associated with reductions, at the genera level, in *Clostridium, Phascolarctobacterium, Serratia, Enterococcus*, *Shigella* and *Shewanella.* Forsberg *et al*. [180] found that administration of *Lactobacillus reuteri* from late pregnancy to one year of age infants decreased IgE-mediated atopic dermatitis at age 2 infants. In a double-blind, prospective, randomized placebo-controlled study of 220 children with atopic dermatitis (ages 1–18), Wang and Wang [181] reported reduced symptoms of atopic dermatitis as well as related altered immune parameters (e.g., IgE and TNF-α) following three-month administration of one or more combinations of *Lactobacillus.* Using a meta-analysis of 25 randomized controlled trials involving administration of *Lactobacillus* species, Kim *et al*. [182] reported probiotic-associated reduction of Scoring of Atopic Dermatitis (SCORAD) values between treatment and control groups across different age groups from one year of age through adulthood.

Probiotic administration of *Bifidobacterium animalis* subsp lactis was found to reduce the puretic symptoms of adult atopic dermatitis [183]. The researchers proposed that anti-puretic metabolites from the probiotic bacterium were responsible for patient improvement. In three separate, randomized, double-blind, placebo-controlled interventions in patients with ulcerative colitis (UC), chronic fatigue syndrome (CFS) and psoriasis, Groeger *et al*. [184] showed that *Bifidobacterium infantis* 35624 displayed beneficial immunoregulatory effects, not just in the gut, but systemically. The plasma pro-inflammatory biomarkers of C-reactive protein (CRP), TNF-α and IL-6 were significantly elevated in patients with all three diseases. CRP was reduced in patients from all three categories, TNF-α was reduced in patients with CFS and psoriasis, and IL-6 was reduced in those with UC and CFS all as compared to placebo. Finally, in a double-blind, placebo controlled study from Finland, Luoto *et al*. [185] found that postnatal administration of prebiotics or probiotics *vs.* placebo to preterm infants (gestational age, ≥32 + 0 and ≤36 + 6 weeks; birth weight, >1500 g) resulted in a significantly lower incidence of clinically-defined, virus-associated respiratory tract infections during the first year of life. Considerable attention has been given to the potential use of probiotics to treat inflammatory bowel disease (IBD) and the results are mixed to date. Part of the likely explanation is that IBD involves two quite different diseases (Crohn’s disease and ulcerative colitis). In a recent meta-analysis, Shen *et al*. [186] concluded that probiotics appear to be most useful in the treatment of ulcerative colitis (probiotics produced elevated remission rates *vs.* placebo).

Probiotics have been used to target extra-gastrointestinal sites as well. Local rebiosis of the vagina with a probiotic formulation following standard azole treatment for *Candida albicans* infection was reported to increase therapeutic efficacy and reduce the relapse rate [187].

Synbiotic administration is a more recent holistic approach to microbiome management than single administration of either a probiotic or prebiotic. For this reason, use of synbiotic formulations is likely to increase as a more targeted alternative to fecal microbiota transplantation. Examples in which treatment with synbiotic formulations have produced reported beneficial results include: the rescue of failure-to-thrive populations of children [188], reduction of postoperative infections among patients undergoing pancreatic surgery [189], useful alterations in total cholesterol, low-density lipoprotein (LDL) cholesterol, and triglycerides and high-density lipoprotein cholesterol and reduction in proinflammatory markers among healthy volunteers receiving a probiotic *Lactobacillus salivarius* UBL S22 and a prebiotic fructo-oligosaccharide *vs*. either placebo or the probiotic alone [190], reduction in proctitis symptoms among prostate cancer patients treated with radiation [191], relief from abdominal pain in irritatable bowel syndrome [192], protection against toxicity in chronic kidney disease [193], and reduction of inflammatory markers in patients with nonalcoholic fatty liver disease [194].

### 7.3 Fecal Microbiota Transplantion

Borody *et al*. [195] recently reviewed the application of fecal microbiota transplantation (FMT) as a strategy for the treatment of both NCDs and infectious diseases. FMT from healthy donors has been used to treat several different conditions. The procedure can involve a colonoscopic fecal transplant [196], administration via a nasogastric tube [197], or non-invasive oral administration of frozen capsules [91]. FMT has been proven to be particularly effective as a therapeutic approach for recurrent *Clostridium difficile* infection [198]. Recently, this approach was adapted for use in pediatric patients [197]. A meta-analysis of the application of FMT to treat inflammatory bowel disease concluded that the treatment was safe, but that there is significant variability among studies in the efficaciousness of the therapy [199]. The authors pointed to the need for better standardization for the selection of donors as well as in the analysis of the microbiome.

To date, FMT has been used primarily for intestinal disorders. However, there is considerable interest in its potential applications for the treatment of extra-gastrointestinal conditions that have been associated with gut microbial dysbiosis. These include metabolic, neurological and immune-associated disorders [200].

In general FMT has been viewed as generally safe [91]. However, it should be noted that safety evaluation of the procedure has been focused on the assessment of short-term adverse outcomes. When colonoscopy is used as the method of fecal transplantation rather than oral administration of frozen capsules, there are colonoscopy-associated risks such as that of peritonitis [201]. Additionally, some transient, self-limiting effects have been noted among recipients such as cramping, fullness, flatulence, bloating, diarrhea and fever [202]. Researchers have called for additional longer-term studies including an evaluation of the potential risk of long-lasting autoimmune disease associated with FMT [203,204].

These examples emphasize both the disease prevention and therapeutic benefits that can result from proactively managing the microbiome whether through prebiotic, probiotic or synbiotic administration or FMT.

## 8. Conclusions

The ongoing epidemic of NCDs combined with increasing antibiotic resistance of bacterial infections has challenged both the integrity of healthcare systems and the global economy. Management of the microbiome across the life stages provides a promising tool against the ongoing epidemic of major healthcare challenges. In effect, prior medical treatments aimed solely at treating our 10% mammalian minority can now be viewed as potentially treating the wrong patient. By focusing disease preventative strategies and medical therapies on the 90% microbial majority of humans rather than solely on our mammalian minority, the goal of personalized medicine can be extended to the entire three-domain-containing human patient. This strategy is not without limitations. Optimization of microbiome treatment regimens awaits further investigation including the potential need to tailor adjustments of microbiota to each life stage, sex, and host genetic background. However, as discussed in this review, our microbiota are at the center of: (1) interactions with the environment, (2) metabolism, and (3) regulation of our host development and physiology. Therefore, a focused priority on microbiome management should increase the effectiveness of risk assessment, drug discovery and efficacy, prevention, and medical therapies bringing us that much closer to sustainable healthcare.
